# The influence of delivery vectors on HIV vaccine efficacy

**DOI:** 10.3389/fmicb.2014.00439

**Published:** 2014-08-22

**Authors:** Beatrice O. Ondondo

**Affiliations:** Nuffield Department of Medicine, The Jenner Institute, University of OxfordOxford, UK

**Keywords:** HIV-1, vaccines, delivery vectors, MVA, NYVAC, adenovirus, ALVAC, DNA

## Abstract

Development of an effective HIV/AIDS vaccine remains a big challenge, largely due to the enormous HIV diversity which propels immune escape. Thus novel vaccine strategies are targeting multiple variants of conserved antibody and T cell epitopic regions which would incur a huge fitness cost to the virus in the event of mutational escape. Besides immunogen design, the delivery modality is critical for vaccine potency and efficacy, and should be carefully selected in order to not only maximize transgene expression, but to also enhance the immuno-stimulatory potential to activate innate and adaptive immune systems. To date, five HIV vaccine candidates have been evaluated for efficacy and protection from acquisition was only achieved in a small proportion of vaccinees in the RV144 study which used a canarypox vector for delivery. Conversely, in the STEP study (HVTN 502) where human adenovirus serotype 5 (Ad5) was used, strong immune responses were induced but vaccination was more associated with increased risk of HIV acquisition than protection in vaccinees with pre-existing Ad5 immunity. The possibility that pre-existing immunity to a highly promising delivery vector may alter the natural course of HIV to increase acquisition risk is quite worrisome and a huge setback for HIV vaccine development. Thus, HIV vaccine development efforts are now geared toward delivery platforms which attain superior immunogenicity while concurrently limiting potential catastrophic effects likely to arise from pre-existing immunity or vector-related immuno-modulation. However, it still remains unclear whether it is poor immunogenicity of HIV antigens or substandard immunological potency of the safer delivery vectors that has limited the success of HIV vaccines. This article discusses some of the promising delivery vectors to be harnessed for improved HIV vaccine efficacy.

## Introduction

Thirty years after the discovery of HIV/AIDS, the search for a safe and effective vaccine has intensified, as a number of promising candidate vaccines progressing to phase IIb/III clinical trials have failed to show efficacy. One of the greatest barriers to HIV vaccine development is the enormous virion diversity (depicted by the existence of numerous clades and subtypes in distinct geographic demarcations) and the continuous evolution which generates numerous quasi-species within an infected individual (Hemelaar et al., 2011). This not only makes it challenging to create immunogens which are effectively matched to the circulating target viruses, but also provides room for immune escape of HIV from potent vaccine-induced immune responses. Therefore, it has emerged that immunogens derived from the most conserved regions of HIV and covering multiple variants (conserved mosaics) stand out as the most suitable candidates for T-cell based vaccines, while immunogens covering the most potent and broadly neutralizing and non-neutralizing antibody epitopes are better for antibody-based vaccines (Emini and Koff, 2004; Robinson and Amara, 2005; McMichael, 2006; Letourneau et al., 2007; Thorner and Barouch, 2007; Sekaly, 2008; Korber et al., 2009; Barouch et al., 2010; Santra et al., 2010; Borthwick et al., 2014). However, the development of a vaccine based on conserved antibody epitopes to provide protective global coverage and to minimize immune escape is hampered by inaccessibility of the highly shielded conserved envelope domains. Furthermore, the observation that development of broadly neutralizing antibodies requires prolonged stimulation with higher antigenic loads from divergent virus species (van Gils and Sanders, 2013) implies that HIV vaccine strategies must provide a continuous high level expression of a cocktail of immunogens. Although the use of polyvalent T-cell and B-cell mosaic constructs or the conserved consensus sequences may effectively overcome the challenges of HIV diversity and significantly improve vaccine efficacy (Santra et al., 2010, 2012), the lack of clearly defined correlates of efficacy means that it remains unclear what immune responses an HIV vaccine should aim to induce. Recently, a non-human primate (NHP) study based on the RhCMV vector induced exceptionally broad and persistent atypical CD8+ T cells which effectively cleared SIV and maintained durable suppression of virus replication (Hansen et al., 2009, 2011, 2013), suggesting that HIV vaccine development research may have to adapt immunogen design and delivery strategies that stimulate similar responses.

Delivery vectors are vital and integral components of a successful vaccine as they play an important role in modulating both innate and adaptive immunity. Therefore, vaccine vectors can significantly influence the magnitude and breadth, as well as the phenotypic and functional qualities of vaccine-induced immune responses. Moreover, as the type of delivery vector, in conjunction with the route of vaccine administration often determine whether or not vaccine-specific immune responses persist within the systemic and/or mucosal compartments (Masopust et al., 2001; Kiyono and Fukuyama, 2004; Ranasinghe et al., 2007; Czerkinsky and Holmgren, 2012), vector choice remains a critical determinant of the overall efficacy of any given vaccine. A part from the immunostimulatory potential to induce strong and persistent immunity, several other factors such as stability and ease of large scale manufacturing, safety, capacity for transgene insertion and pre-existing immunity also influence vector choice. It is now well-documented that pre-existing anti-vector immunity (especially neutralizing antibodies) can prevent transduction and/or expression of vaccine transgenes thus reducing vaccine-specific immune induction (Xiang et al., 2002; Fitzgerald et al., 2003; Lasaro and Ertl, 2009). This is a common phenomenon, clearly demonstrated with certain vectors which show superior immunogenicity in animal models yet induce only modest immune responses due to neutralization by pre-existing antibodies in humans (McCoy et al., 2007). Additionally, pre-existing immunity can alter the natural course of infection leading to catastrophic consequences such as enhanced HIV acquisition and possibly accelerated disease progression (Buchbinder et al., 2008; McElrath et al., 2008). Thus strategies that concurrently maximize vaccine immunogenicity while minimizing safety concerns remain an urgent priority in the development of a safe and efficacious vaccine for HIV/AIDS.

A good number of HIV vaccine candidates (both prophylactic and therapeutic) employing a broad range of vaccine delivery vectors have been tested and some have progressed to evaluation of potential efficacy in phase IIb/III trials. Of significant relevance as far as safety is the STEP trial that used human adenovirus serotype 5 (Ad5) to deliver a well-designed HIV immunogen expressing Gag/Pol/Nef, which was associated with increased risk of HIV acquisition in uncircumcised male vaccinees with pre-existing immunity to Ad5 (Buchbinder et al., 2008; McElrath et al., 2008). This unexpected and rather worrisome finding prompted the premature halting of two related efficacy trials due to futility (Gray et al., 2011; Hammer et al., 2013). As disappointing as this might have been at the time, invaluable lessons have been learned and there is still great optimism as these lessons are now taken on board. Focussing on some of the promising HIV vaccine candidates in preclinical and clinical development, this review discusses pertinent issues relating to safety and immunogenicity of replicating and non-replicating viral vectors, pre-existing anti-vector immunity and how these can potentially influence the natural history of HIV infection and progression. In particular, this article highlights the safety profiles, immuno-stimulatory potential and possible limitations of plasmid DNA, MVA (modified vaccinia virus Ankara), ALVAC (canarypox virus), NYVAC (New York attenuated vaccinia virus), influenza virus and adenovirus vectored vaccines in preclinical and clinical studies for HIV vaccines. Some of the delivery vectors evaluated in clinical studies are summarized in Table 1, while those in preclinical development are summarized in Table 2.

**Table 1:** **Representative clinical studies**.

**Study name and phase**	**Immunogen**	**Vectors, regimen and route of immunization**	**Immune responses generated**	**References**
**(I) HETEROLOGOUS PRIME-BOOST STUDIES**				
HIVCORE002 (Phase I study)	HIVconsv (T cell immunogen based on conserved regions)	ChAdV63/MVA (i.m.)	-CD4+ and CD8+ T cells -*In vitro* virus inhibition	Borthwick et al., 2014
		DNA/ChAdV63/MVA (i.m.)		
		DNA/MVA/ChAdV63 (i.m.)		
HVTN 505 (Phase IIb study)	VRC-HIVDNA016-00-VP/VRC-HIVADV014-00-VP	DNA-prime (i.m. biojector device)/rAd5 boost (i.m. needle and syringe)	-T cells and gp140 binding IgG antibodies	Hammer et al., 2013
HVTN 503/Phambili (Phase IIb study)	MRKAd5 HIV-1 Gag/Pol/Nef	DNA-prime (i.m.)/Ad5 boost (i.m.)	-CD8+ and CD4+ T cells	Gray et al., 2011
Phase 1 study	Gag and Env DNA and recombinant trimeric Env glycoprotein	DNA-prime (i.m.)/Protein boost with MF59 adjuvant	-Robust B and T cells	Spearman et al., 2011
			-Strong NAbs to SF162	
			-ADCC and neutralization of tier 2 strains	
Phase I/II study	Multi-clade, multigene: DNA/HIV-1 gp160, p17/p24 Gag and MVA/HIV-1 Gag/Pol	Low dose (i.d.) DNA-prime (x3)/MVA-boost (i.m. x2)	-High magnitude and broad CD4+ and CD8+ T cell responses	Bakari et al., 2011
		(DDDMM)	-Env antibodies	
Phase I study DP6-001	Multigene polyvalent gp120 and Gag DNA and polyvalent gp120 protein	i.m. or i.d. Polyvalent DNA-prime/i.m. protein-boost (with QS21 adjuvant)	-High titer binding and BNAbs, ADCC and multifunctional T cells	Bansal et al., 2008; Vaine et al., 2010
RV144 (Phase III study)	ALVAC-HIV vCP1521/AIDSVAX gp120 B/E	ALVAC-prime (i.m.)/gp120 protein-boost	-T cells and non-neutralizing antibodies to V1/V2 loop	Rerks-Ngarm et al., 2009
Phase I study	Multigenic HIV DNA (gp160- A/B/C; Rev B, Gag A/B and RT- B and HIV-MVA Env/Gag/Pol)	DNA- prime (i.d. with Biojector)/MVA-boost (i.d./i.m.); with or without GM-CSF adjuvant	-Broad and potent cellular immune responses	Sandstrom et al., 2008; Gudmundsdotter et al., 2009
HVTN 502/STEP Study (Phase IIb)	MRKAd5 HIV-1 Gag/Pol/Nef	DNA-prime (i.m.)/Ad5 boost (i.m.)	-Strong CD8+ T cell responses	Buchbinder et al., 2008; McElrath et al., 2008
Phase 1 study	HIVA (HIV-1 clade A and a CTL epitope string)	DNA-prime (i.m.)/MVA-boost (i.m.)	-Multifunctional CD4+ and CD8+ T cells	Mwau et al., 2004; Goonetilleke et al., 2006
Phase I study (EuroVacc: EV02)	HIV-1 clade C-Env/Gag/Pol/Nef (DNA-C and NYVAC-C)	DNA-prime (i.m.)/NYVAC- boost (i.m.)	-Durable, broad and poly-functional CD4+ and CD8+ T cells	Harari et al., 2008; McCormack et al., 2008
Phase I study	ALVAC-HIV(vCP300)	ALVAC-prime (i.m.)/i.m. Protein-boost (with MF59 adjuvant)	-Durable CTLs	Evans et al., 1999
	gp120/gp41, Gag, Pro, Nef, Pol and SF-2 rgp120		-Antibody responses	
Phase I study	ALVAC-HIV(vCP205)	ALVAC-prime (i.m.)/i.m. Protein-boost (with MF59 adjuvant)	-Strong CD8+ T cell responses and NAbs	Belshe et al., 1998
	gp120/gp41, Gag, Pol and SF-2 rgp120			
**(II) HOMOLOGOUS PRIME-BOOST OR SINGLE DOSE STUDIES**				
HVTN-070 and -080 Phase I studies	PV (PENNVAX(R)-B DNA expressing Gag, Pol, Env and DNA/IL-12	DNA+IL-12 (i.m. or by electroporation)	-CD4+ and CD8+ T cell responses	Kalams et al., 2013
IPCAVD-001	Ad26.ENVA.01	Intramuscular delivery of rAd26	-Binding antibodies	Baden et al., 2013; Barouch et al., 2013
			-Multiple CD8+ and CD4+ T cell responses	
			-ADCC and virus inhibition	
HVTN 090 Phase Ia study	VSV_IN_N4CT1_Gag_1 (recombinant VSV expressing HIV-1 Gag)	Dose-escalating i.m. delivery	Low level T cell responses detected following initial dosing	Fuchs et al., 2012, 2013
Phase I study	Ad35-GRIN (Gag, RT, Integrase, Nef) and Ad35-GRIN/ENV	Intramuscular delivery of Ad35-GRIN/Env or Ad35-GRIN	-Robust, broad and polyfunctional CD4 and CD8+ T cells	Keefer et al., 2012
Phase I/II study (RISVAC02)	MVA-B (monomeric gp120 and clade B Gag/Pol/Nef poly-protein)	Three doses of MVA (i.m.)	-Durable antibody and cellular immune responses	Garcia et al., 2011; Gomez et al., 2011
Phase I study	ADVAX (multigenic HIV-1 DNA vaccine)	DNA by i.m. electroporation	-CD4 and CD8+ T cells with multiple cytokines	Vasan et al., 2011
VAX 003 (Phase III study)	Bivalent recombinant gp120 vaccine: AIDSVAX B/E	Seven i.m. injections; with Alum adjuvant	-Binding and neutralizing antibodies to gp120	Pitisuttithum et al., 2006
VAX 004 (Multicentre Phase III study)	Bivalent recombinant gp120 vaccine: AIDSVAX B/B	Seven i.m. injections; with Alum adjuvant	-Binding and neutralizing antibodies to gp120	Flynn et al., 2005; Gilbert et al., 2005

*i.m., intramuscular; i.n., intranasal; i.d., intradermal; s.c., subcutaneous; i.p., intraperitoneal; ADCC, antibody dependent cytotoxicity; NAbs, neutralizing antibodies; BNAbs, broadly neutralizing antibodies*.

**Table 2:** **Representative preclinical studies**.

**Animals**	**Immunogen**	**Vectors, regimen and route of immunization**	**Immune responses generated**	**Outcomes**	**References**
**(I) HETEROLOGOUS PRIME-BOOST STUDIES**					
Mice and rabbits	HIV Env/Gag-Pol-Nef DNA, MVA-C (HIV Env/Gag-Pol-Nef and CN54gp140 protein)	Intramuscular delivery of DNA/MVA/Protein with TLR4 (GLA-AF adjuvant) for protein boost	Antibody and T cell responses	–	McKay et al., 2014
Rhesus macaques	SIVmac239 Env/Gag DNA, rmIL-12 DNA and SIVmac239 protein vaccines	DNA-prime (by electroporation)/i.m. or i.d. Protein-boost, or DNA and protein co-immunization	Persistent mucosal Envelope-specific antibody responses	Enhanced immunity by the co-immunization modality	Jalah et al., 2014
Rhesus macaques	SIV-Gag mosaic	DNA-prime (x3, i.m.) Ad5-boost (i.m.)	-NAbs	Protection against SIVsmE660 challenge	Roederer et al., 2014
	SIV-Env mosaic		-ADCC		
	SIV_mac239_ Env		-Cellular responses		
Rhesus monkeys	DNA expressing SIVmac239 antigens + rmIL-12 and inactivated SIVmac239 virus particles as protein	DNA prime (i.m. followed by *in vivo* electroporation) /protein-boost	-SIV-specific CTLs	-Protection from SIV_SM_E660 acquisition	Patel et al., 2013
			-CD4+ and CD8+ memory T cells		
		-Binding antibodies	-Reduced peak and chronic phase viremia		
Mice	pCCMp24	DNA prime/Tiantan boost (i.m.)	Antibody and T cells	–	Excler et al., 2010; Liu et al., 2013
	rddVTT-CCMp24				
Rhesus macaques	SIV_SME543_-Gag/Pol/Env	Prime-boost (i.m.) with: Ad26/MVA, Ad35/Ad26, DNA/MVA, MVA/Ad26	-NAbs	Protection from SIVmac251 acquisition or disease progression	Barouch et al., 2012
			-Binding antibodies		
			-Cellular responses		
Mice	Ad35-GRIN/ENV and MVA-C (Gag/Env/Pol)	Ad35-GRIN/ENV-prime (i.m.)/MVA-boost (i.m.)	Polyfunctional CD8+ T cells	–	Ratto-Kim et al., 2012
Macaques	SIV DNA/GM-CSF (SIV239 Gag/PR/RT/Env/Tat/Rev) and MVA-SIVgpe	DNA/GM-CSF- prime (i.m.)/MVA-boost (i.m.)	-Neutralizing antibody responses	Sterile protection after SIVsmE660 challenge	Lai et al., 2011
			-ADCC		
Murine	DNA-Env and gp120 protein vaccines	DNA Env-prime/gp120 protein-boost (i.m. and i.n.)	-Persistent mucosal and systemic Abs	–	Cristillo et al., 2011
		(Advax-M and Advax-P adjuvants)	-T cell responses		
New-born and adult mice	BCG-HIVA, MVA-HIVA and HAdV5.HIVA	BCG-prime (i.p./i.d./s.c.) followed with i.m. MVA- or HAdV5- boost	-Strong, cytotoxic CD8+ T cell responses	–	Hopkins et al., 2011a; Saubi et al., 2011
Rhesus macaques	VSV and SFV replicon expressing SIV-Gag/Env	VSV-prime (i.m. and i.n.)/SFVG-boost (i.m.)	-High titer NAbs to Env proteins and weak cellular responses	-Sterilizing immunity Control of SIVsmE660 breakthrough infections	Schell et al., 2011
New-born macaques	VSV-SIVgpe (rVSV- Gag/Pol/Env) and MVA-SIVgpe	VSV-prime (oral)/MVA-boost (i.m.)	-Systemic Abs, both systemic and local cellular responses	–	Van Rompay et al., 2010
Mice, rabbits and macaques	Consensus or Polyvalent mosaic DNA and protein (gp120) vaccines	DNA-prime (i.m.)/i.m. and i.d. rVaccinia-boost.	-Broadly neutralizing antibodies and CD8+ T cell responses	Enhanced immunogenicity	Wang et al., 2006b; Santra et al., 2010
		DNA-prime (gene gun)/ Protein-boost (i.d.) + IFA			
Rhesus macaques	VSV-SHIVGag/Pol/Env	VSV-prime (i.m.)/MVA-boost (i.m.)	-Persistent multi-functional	Durable (over 5 years) control of SHIV89.6P replication	Rose et al., 2001 Schell et al., 2009
	MVA-SHIVGag/Pol/Env		CD8+ T cells and NAbs		
Rabbits macaques	HIV-1 Env gp120	DNA (electroporation)/gp120 protein boost	-Persistent Th1, CTL and Env responses	Neutralization of sensitive SHIV isolates	Cristillo et al., 2008
Rhesus macaques	CMV-SHIVdEN and SeV-Gag	DNA prime (i.m.)/Sendai Virus boost (i.n.)	-CD8+ T cells	Durable control of SIVmac239 and SHIV89.6PD	Matano et al., 2001; Takeda et al., 2003; Kawada et al., 2007
Rhesus Macaques	replication-defective SHIV particles and MVA-SHIV (SIV Gag, SIV Pol and HIV Env)	Intrarectal DNA prime/MVA boost	-Antibodies in plasma	-Preserved CD4 T cells -Reduced disease progression after SHIV 89.6P challenge	Wang et al., 2004
			-Cellular responses		
Rhesus macaques	SHIV-DNA plus IL-2 and rMVA	DNA + IL-12-prime (i.n.)/MVA-boost (i.n.)	-Mucosal and systemic antibody and cellular responses	Protection from SHIV 89.6P challenge	Bertley et al., 2004
Mice and monkeys	E1/E3-deleted AdHu5 and E1-deleted AdC7 or AdC6, expressing Gag37	i.m. prime-boost with: AdC7/AdC6/AdHu5 or AdHu5/AdC6/AdC7	-Robust CD8+ CD4+ T cells	–	Reyes-Sandoval et al., 2004
			-Antibody responses		
Cynomolgus macaques	DNA- HIV-1 IIIB Env/Gag/RT/Rev/Tat/Nef, MVA- HIV-1 IIIB Nef-Tat- Rev, SIVmacJ5 Gag/Pol and Vaccinia HIV-1 Env	DNA prime/MVA boost (i.m. or mucosally)	-Antibody and cellular responses	Protection from infection	Makitalo et al., 2004
Mice	HIV-1 Env IIIB Ag (DNA-Env and MVA-Env)	DNA-Env-prime/MVA-Env-boost (i.n. with Cholera toxin adjuvant)	-Mucosal CD8+ T cells, mucosal and systemic antibodies	–	Gherardi et al., 2004
			-Beta-chemokines		
Rhesus monkeys	DNA, MVA and Ad5 vectors expressing SIVmac239 Gag	DNA Prime (i.m.)/MVA- or Ad5- boost (i.m.)	-Robust CD8+ T cells with cytotoxic activity	Pronounced attenuation of SHIV infection and mitigated disease progression	Shiver et al., 2002
Macaques	DNA and NYVAC SIV-gpe (Gag/Pol/Env)	DNA-prime (i.m.)/NYVAC-boost (i.m.)	-Durable CD8+ T cell responses	–	Hel et al., 2001
**(II) HOMOLOGOUS PRIME-BOOST OR SINGLE DOSE STUDIES**					
Mice and rabbits	Ad4Env160	i.m., i.n., or s.c. delivery of rAd4	-T cell and antibody responses	Neutralization of tier-1 and tier-2 pseudoviruses	Alexander et al., 2013
	Ad4Env140				
	Ad4Env120				
Mice	Ad35-GRIN/ENV and MVA- Gag/Env/Pol	Ad35-prime (i.m.)/Ad35-boost i.m.): MVA-prime (i.m.)/MVA-boost (i.m.)	-Polyfunctional CD8+ T cells	–	Ratto-Kim et al., 2012
Rhesus macaques	SIV_SME543_-Gag/Pol/Env	MVA-prime (i.m.)/MVA-boost (i.m.)	-Neutralizing Abs, binding antibodies and cellular responses	Protection from SIVmac251 acquisition or disease progression	Barouch et al., 2012
Rhesus macaques	RhCMV-SIV/Gag, Rev/Nef/Tat, Pol, Env	RhCMV vectors delivered by s.c. injection	-Strong and persisting, polyfunctional effector memory CD8+ and CD4+ cells	Viral clearance and durable protection from SIVmac239 disease progression	Hansen et al., 2009, 2011
Rhesus monkeys	SIV-Gag, SIV-Env and SIV Rev-Tat-Nef fusion protein	Intravenous delivery of recombinant Rhadinovirus	-Persistent effector memory CD8+ T cells	Control of SIVmac239 replication	Bilello et al., 2011
Rhesus macaques	Rabies virus (RV) expressing SIVmac239 Gag/Pol or Env	Intramuscular delivery of rRV constructs	-Polyfunctional CD8+ T cells in the mucosa	Control of SIVmac251-CX challenge	Faul et al., 2009
			-NAbs		
Rhesus and Cynomolgus macaques	SIV-Gag DNA + rIL-12 DNA vaccines	Intramuscular DNA delivery	T cell and Antibody responses	Improved clinical outcome after SHIV[89.6P] challenge	Boyer et al., 2005; Chong et al., 2007
Juvenile and Infant Rhesus macaques	ALVAC-SIV and MVA-SIV both expressing SIV-Gag/Pol/Env	Multiple immunizations with ALVAC-SIV (i.m.) or MVA-SIV (i.m.)	-High titres of binding antibodies, low-level T cell responses	Protection from oral SIVmac251 challenge, and reduced viremia in breakthrough infections	Van Rompay et al., 2005
Mice	HIV-1 Env IIIB Ag (DNA-Env and MVA-Env)	MVA-Env/MVA-Env	-Mucosal CD8+ T cells, mucosal and systemic antibodies	–	Gherardi et al., 2004
		DNA-Env/DNA-Env (i.n. with Cholera toxin adjuvant)	-Beta-chemokines		
Mice	Influenza virus expressing HIV-1 ELDKWA epitope	i.n. prime/boost with chimeric influenza virus, followed with i.p. boost with live virus	-Neutralizing antibodies	Neutralization of distantly related HIV-1 isolates	Muster et al., 1994

*i.m., intramuscular; i.n., intranasal; i.d., intradermal; s.c., subcutaneous; i.p., intraperitoneal; ADCC, antibody dependent cytotoxicity; NAbs, neutralizing antibodies; BNAbs, broadly neutralizing antibodies*.

## Recombinant DNA vaccine vectors

DNA plasmid vaccines can induce both T and B cell immune responses, and are popular for their safety, stability, versatility and ease of large scale production. Most importantly is the fact that they can be used repetitively to boost immunity (Valentin et al., 2010) without the risk of immune interference as is the case with viral vectors with high prevalence of pre-existing immunity. However, on their own DNA plasmid vaccines have exhibited very limited immunostimulatory capacity and often induced sub-optimal immune responses. Recent advances in DNA delivery such as intramuscular, skin or intradermal electroporation (Selby et al., 2000; Widera et al., 2000; Brave et al., 2010; Vasan et al., 2011; Kopycinski et al., 2012) or use of other physical delivery methods such as gene gun and biojector devices (Drape et al., 2006; Wang et al., 2008a; Graham et al., 2013), together with concurrent use of cytokine adjuvants including IL-2, IL-12, and IL-15 (Winstone et al., 2011; Kalams et al., 2012, 2013) have greatly improved the immunogenic potential of DNA vaccines. In particular, IL-12 was shown to significantly augment the frequency, magnitude and breadth of Gag-specific immune responses in healthy volunteers immunized with a recombinant DNA vaccine expressing HIV-1 Gag (Kalams et al., 2012, 2013). Similarly, when macaques were co-immunized with a plasmid encoding IL-12 and a DNA plasmid expressing SIV-Gag, strong antibody and cellular responses which correlated with a better clinical outcome were induced (Boyer et al., 2005; Chong et al., 2007). More impressively, co-delivery of a plasmid encoding GM-CSF with a DNA vaccine expressing SIV genes induced strong neutralizing antibody responses and ADCC, which protected against infection with SIVsmE660 (Lai et al., 2011). The use of strong adjuvants such as glucopyranosyl lipid A (a TLR4 agonist) in a DNA/MVA/protein immunization regimen was shown to enhance both antibody and T cell responses (McKay et al., 2014), while plasmids encoding the TLR5 agonist, flagellin, enhanced both antibody and T cell immunity to influenza virus (Applequist et al., 2005)

Other significant improvements in DNA vaccine technology include codon optimization, use of stronger promoters/enhancers and signal peptides such as the tissue plasminogen activator (tPA) and lysosome associated membrane protein (LAMP1), all of which significantly enhance transgene expression and trafficking, thus leading to increased vaccine immunogenicity (Wang et al., 2006a; Yan et al., 2007; Wallace et al., 2013). Furthermore, ease of DNA manipulation provides a platform to deliver polyvalent or multi-gene vaccine components which can increase the breadth and depth of vaccine-induced immunity to reduce immune escape. This strategy showed remarkable success in rabbit experiments where a polyvalent gp120 vaccine induced broadly neutralizing antibody responses as opposed to the monovalent vaccine (Wang et al., 2006b). Similarly, polyvalent mosaic plasmid DNA vaccines have demonstrated enhanced immunogenicity in mice (Kong et al., 2009) and rhesus monkeys (Santra et al., 2010).

Several studies indicate that delivery of DNA vaccines by electroporation induces both cellular and humoral immune responses which are long-lived and can persist for several years with or without subsequent heterologous boosting (Cristillo et al., 2008; Patel et al., 2010; Jalah et al., 2014). In particular, the level of HIV-specific immune responses to the multigenic ADVAX vaccine was increased by up to 70-fold when electroporation was used for delivery (Vasan et al., 2011). Nonetheless, DNA vaccines consistently show much better immunogenicity when used as priming components in conjunction with viral vectors such as adenoviruses (Shiver et al., 2002; Hammer et al., 2013; Borthwick et al., 2014), MVA (Sandstrom et al., 2008; Gudmundsdotter et al., 2009; Bakari et al., 2011; Borthwick et al., 2014), fowlpox (Kent et al., 1998), and NYVAC (Hel et al., 2001) in heterologous prime boost regimens delivering the same vaccine inserts, or in co-immunization strategies that combine DNA-prime with protein boosting (Kennedy et al., 2008; Wang et al., 2008b). As a matter of fact, prime-boost regimens still remain the most successful strategies that emphasize the potential of DNA vaccines. It was recently shown that a DNA-prime/protein-boost regimen was significantly better than either DNA/DNA or protein/protein alone regimens for generating long-term protection of mice against *Leishmania donovani* (Mazumder et al., 2011). The DNA and protein co-immunization modalities are particularly desirable as they maximize induction of long-lived humoral and cellular immune responses which can disseminate to mucosal sites, including the genito-rectal mucosae (Patel et al., 2013; Jalah et al., 2014). A recent study has demonstrated in small animal models that concurrent, multiple-route DNA vaccinations comprising DNA prime by electroporation, followed with intranasal, intramuscular, subcutaneous or transcutaneous homologous protein boost induced strong HIV-specific B and T cell responses (Mann et al., 2014). Independently, another study showed enhancement of HIV gp120-specific IgA responses in serum and mucosal secretions following a DNA env-prime and gp120 protein-boost delivered with novel carbohydrate-based adjuvants (Advax-M and Advax-P) which were specifically designed for mucosal and systemic immune enhancement (Cristillo et al., 2011). The tremendous effect of a DNA prime in enhancing antibody responses to protein vaccines was also documented in a Phase 1 clinical study, where intramuscular delivery of a DNA priming vaccine followed with recombinant protein boost stimulated higher frequencies of B and T cells, as well as higher neutralizing antibody titres and ADCC in contrast to immunization with protein alone (Spearman et al., 2011). Perhaps the most exciting of the DNA-prime/protein-boost studies is the 6-plasmid polyvalent DNA vaccine expressing gp120 and Gag, followed by QS21-adjuvanted polyvalent gp120 protein boost (DP6-001 study) in which multifunctional T cells and high-titre gp120-specific binding and broadly-neutralizing antibodies as well as ADCC were induced (Graham et al., 2006; Bansal et al., 2008; Wang et al., 2008b; Vaine et al., 2010).

Apart from effective delivery strategies and routes of immunization, there is evidence showing that expression of DNA vaccines and subsequent immunogenicity in humans and other primates can be limited by serum amyloid P component (SAP), a protein found in blood and known to bind strongly to DNA (Wang et al., 2011, 2012). In small animals this protein either binds weakly or does not exist at all. Thus, depletion of SAP protein prior to administration of DNA vaccines is another new strategy being tested to improve DNA vaccine immunogenicity. This concept has been proven in mice, where depletion of SAP using the bis-d-proline compound CPHPC (Bodin et al., 2010; Gillmore et al., 2010) was shown to augment antibody and cellular immune responses to a DNA vaccine expressing Hepatitis B surface antigens (Wang et al., 2012). The concept is currently being tested in a Phase 1 clinical trial (HIVCORE003) of healthy adults using the T-cell based HIV candidate vaccine, HIVconsv.

Although the efficacy of an HIV DNA vaccine is yet to be demonstrated in humans, various studies (prophylactic and therapeutic) in the macaque model have reported protective immune responses which controlled SIV/SHIV replication or protected from infection (Rosati et al., 2005, 2009; von Gegerfelt et al., 2007; Valentin et al., 2010; Patel et al., 2013). In particular, a study combining a DNA/MVA mucosal delivery of a DNA construct expressing replication-defective SHIV particles and MVA expressing SIV-Gag/Pol and HIV Env (MVA-SHIV) demonstrated significant protection from disease progression after a SHIV89.6P challenge (Wang et al., 2004). Furthermore, mucosal co-delivery of a DNA priming vaccine together with an IL-2 encoding vector, followed by MVA boost also induced protective immunity against SHIV89.6P challenge (Bertley et al., 2004). The results in these macaque models, together with the documented efficacy of DNA vaccines against animal diseases [e.g., equine West Nile Virus (WNV) (Davis et al., 2001), melanoma in dogs (Bergman et al., 2003) and infectious hematopoietic necrosis virus (IHNV) in salmon (Garver et al., 2005; Kurath et al., 2006)] raise hopes that with the right immunogen and effective delivery strategies (including adjuvants), plasmid DNA vaccines for HIV/AIDS could achieve efficacy in clinical trials, when used alone, but more realistically in prime-boost combinations with live viral-vectored or protein vaccines.

## Non-replicating recombinant viral vectors

### Adenovirus vaccine vectors

Adenoviruses are the most powerful vectors for inducing both antibody and cell-mediated immunity to inserted transgenes and are known to elicit between 5- and 10-fold stronger T cell responses compared to conventional naked DNA or MVA/pox-like virus vectors (Xiang et al., 1996; He et al., 2000; Fitzgerald et al., 2003; Casimiro et al., 2003a, 2004; Tatsis and Ertl, 2004; Catanzaro et al., 2006). The Adenovirus vectors use either the Coxsackie and Adenovirus Receptor (CAR) or CD46 receptors (Bergelson et al., 1997; Gaggar et al., 2003) and can infect a wide variety of cells, including dendritic cells. In particular, group B adenoviruses such as Ad35 recognize CD46 surface protein and infect DCs more efficiently than group C isolates. These vectors achieve higher levels of transgene expression which in turn results in stronger and persistent immune effector functions (Zhang et al., 2001; Hutnick et al., 2010; Suleman et al., 2011). Several studies indicate that adenoviruses predominantly stimulate persistent effector memory CD8+ T cell responses (Yang et al., 2003a, 2007a; Tatsis et al., 2007a) which are more suitable for immediate control of invading pathogens at peripheral entry sites such as the genital mucosa (Cerwenka et al., 1999; Sallusto et al., 2004; Huster et al., 2006), and have shown tremendous success in animal studies (Liu et al., 2009). In addition to the effector memory T cells, stable central memory CD8+ T cell populations are also generated, thus providing surveillance in both peripheral and lymphoid sites. Although persisting adenovirus-driven immune responses could also be due to the long-term presentation of antigens by non-haematopoietic cells serving as unlimited antigen depot (Finn et al., 2009; Kim et al., 2010; Bassett et al., 2011), long-lived immunity is largely attributed to persisting low-level expression of inserted immunogens. Adenovirus genomes are known to persist for prolonged periods in various cell types (including those at inoculation sites) where they remain transcriptionally active and continuously produce low-levels of antigen to prime naïve T cells while also maintaining the effector memory T cells (Yang et al., 2006, 2007b; Tatsis et al., 2007a). Furthermore, the arising effector memory T cells express the IL-7 receptor (CD127) which allows their prolonged survival in the absence of antigen. Besides induction of potent adaptive immune responses, adenoviruses also stimulate innate immunity via highly inflammatory responses which involve TLR2, TLR9, NOD-like receptors and the type 1 interferon pathways that result in abundant cytokine and chemokine secretion (Hensley et al., 2005; Nazir and Metcalf, 2005; Appledorn et al., 2008; Muruve et al., 2008). Another attractive feature of adenovirus vectors is their ability to induce both systemic and mucosal immune responses following parenteral delivery, as well as their suitability for mucosal immunization (Sharpe et al., 2002; Xiang et al., 2003; Bangari and Mittal, 2006; Haut et al., 2010).

The most well-characterized of the adenovirus vectors is human Ad5, successfully used as a delivery vector for a rabies vaccine and found to be very good at inducing protective virus neutralizing antibodies concurrently with CD8+ and CD4+ T cells (Xiang et al., 1995, 1996). In the HIV field, Ad5 was used as a booster immunization following DNA priming and induced strong CD8+ T cell responses in a large proportion of the STEP study vaccinees (Buchbinder et al., 2008; McElrath et al., 2008). However, clinical efficacy may have been significantly compromised by pre-existing neutralizing antibodies (ranging from 40 to 70% in developed countries and greater than 90% in developing countries) and cellular immunity (Fitzgerald et al., 2003; Holterman et al., 2004; Bangari and Mittal, 2006; Xiang et al., 2006; Lasaro and Ertl, 2009; Ersching et al., 2010; Mast et al., 2010; Barouch et al., 2011). These results were recapitulated in a non-human primate study using low-dose penile exposure to SIVmac251 in Ad5 seropositive animals immunized with SIV-Gag/Pol/Nef (Qureshi et al., 2012). Possibly, adenovirus vaccination boosted the numbers of activated CD4+ T cells which are targets for HIV-1 (Benlahrech et al., 2009). While this might seem a plausible explanation, especially when considering the potential of such activated targets to traffic to the genito-rectal mucosae (Tatsis et al., 2007a; Benlahrech et al., 2009), this argument is strongly contested by observations that other vaccine carriers such as DNA and MVA do stimulate CD4+ T cell activation but have not been associated with increased HIV acquisition. However, it is worth noting that DNA/MVA vaccines are yet to be tested for efficacy in large clinical trials and as such their potential to enhance HIV acquisition has never assessed. Furthermore, DNA/MVA vaccines combinations have not been associated with long-term persistence of activated T cells or mucosal homing. Another postulated theory is the formation of adenovirus-specific antibody immune complexes that activate both dendritic and CD4+ T cells hence fuelling infection (Perreau et al., 2008). In this study, Ad5 immune complexes were strongly correlated with higher HIV infection in the *in vitro* cultures, thus supporting a stronger likelihood of enhanced HIV acquisition. Should either or both of these theories be true, this would have dire consequences for other clinical trials using Ad5 to deliver non-HIV immunogens such as malaria (Sedegah et al., 2011; Tamminga et al., 2011; Chuang et al., 2013) and TB (Smaill et al., 2013) vaccines which will induce similar phenotypes and pre-dispose the vaccinees to increased HIV acquisition risk, although this may not be apparently detectable as these studies may not monitor HIV acquisition.

Apart from the issue of pre-existing immunity, immunization with Ad5 can induce neutralizing antibodies in naïve individuals which can be a hindrance for successive immunizations with the same or cross-reactive adenoviral vectors (Casimiro et al., 2003b; Bangari and Mittal, 2006). Thus new rare adenovirus vectors with lower pre-existing immunity such as Ad26 and Ad35 are becoming more attractive (Holterman et al., 2004; Abbink et al., 2007; Barouch et al., 2012; Zhang et al., 2013), although these are relatively less immunogenic compared to Ad5 (Colloca et al., 2012). Besides the lower sero-prevalence, Ad26 neutralizing antibody titres are usually very low compared to Ad5 (Abbink et al., 2007; Chen et al., 2010; Mast et al., 2010). As an HIV vaccine delivery vector, Ad26 was shown to induce broadly functional cellular and antibody responses with viral inhibitory capacity in a first-in-human (IPCAVD-001) clinical trial of an HIV envelope immunogen (Ad26.ENVA.01) (Baden et al., 2013; Barouch et al., 2013). In this study, a dose-dependent expansion of the magnitude, breadth, and epitopic diversity of Env-specific binding antibody responses were observed. The responses comprised multiple CD8+ and CD4+ T cell memory subpopulations and cytokine secretion phenotypes. Antibody-dependent cell-mediated phagocytosis and degranulation functional activity were also observed. Ad35 has also shown high immunogenicity in healthy volunteers, eliciting robust and polyfunctional CD8+ and CD4+ T cells in a majority of volunteers immunized with Ad35-GRIN (an immunogen based on Gag, RT, integrase and nef) or Ad35-GRIN/ENV (premixed Ad35-GRIN and Ad35-ENV vaccines) (Keefer et al., 2012). Similarly, in BALB/c mice, an Ad35-GRIN/ENV-prime followed by a boost with rMVA containing Gag/Env/Pol genes from various HIV-1 clades induced polyfunctional CD8+ Gag-specific central and effector memory T cells which were superior to those elicited in homologous Ad35/Ad35 or MVA/MVA prime boosts (Ratto-Kim et al., 2012).

Other rare adenovirus vectors include human Ad6, chimpanzee Ad3, Ad63, and Ad68 (Barnes et al., 2012; Colloca et al., 2012; Dicks et al., 2012; O'Hara et al., 2012; Roshorm et al., 2012). The chimpanzee adenoviruses remain attractive in particular due to their high immunological potency and low sero-prevalence, as well as extremely low or virtually absent cross-reactivity with human adenoviruses (Xiang et al., 2006; Chen et al., 2010; Colloca et al., 2012). Furthermore, chimpanzee adenoviruses induce stronger T and B cell responses in heterologous prime-boost regimens even in the presence of pre-existing immunity to Ad5 (Tatsis et al., 2009). Apart from using these naturally occurring human and chimpanzee adenoviruses, new derivatives of adenovirus vectors that have equivalent immunogenicity but with significantly lower pre-existing antibodies are currently being developed (Dicks et al., 2012; Lopez-Gordo et al., 2014). However, it is worth noting that pre-existing cellular immunity (CD8+ and CD4+ T cells) may be a major deterrent as unlike antibodies, these cells are highly cross-reactive across adenovirus serotypes because they are directed to conserved sequences of adenovirus (Olive et al., 2002; Fitzgerald et al., 2003; Frahm et al., 2012). Nevertheless, some studies indicate that Ad5 and Ad26 vectors can still elicit significant systemic and mucosal responses even in people with pre-existing immunity (Barouch et al., 2013; Smaill et al., 2013). Immunogenic adenoviruses faced with significant pre-existing immunity problems can be improved by modification of the antibody-binding sites, especially within the variable hexon loops in order to reduce NAb binding whilst maintaining immunogenicity (Bruder et al., 2012). This can be achieved via point mutations or complete replacement (Roberts et al., 2006; Abe et al., 2009; Pichla-Gollon et al., 2009; Bruder et al., 2013).

Besides their immunogenicity when used alone, adenovirus vaccines are also very immunogenic when used to prime responses which are then boosted by other vaccine vectors (Tatsis et al., 2007b; Ratto-Kim et al., 2012). In particular, adenovirus-prime followed with MVA-boost can induce high frequencies of much more long-lived, potent T cells (Reyes-Sandoval et al., 2008, 2010; Capone et al., 2010; Hill et al., 2010). A Phase I clinical trial of a T-cell HIV vaccine based on the conserved regions was recently shown to elicit exceptionally high magnitude and polyfunctional T cell responses (circa 5000 IFN-γ ELISPOT SFU/million cells) in HIV-negative healthy volunteers when primed with chimpanzee Ad63 (ChAdV63-HIVconsv) followed with MVA-HIVconsv boost (Borthwick et al., 2014). The vaccine-induced CD8+ T cells exhibited potent *in vitro* antiviral activity. This study also demonstrated that the magnitude and functional capacity of T cells induced in a regimen comprising three priming doses of DNA followed with ChAdV63 and MVA (DDDCM) did not differ significantly from those in a simplified ChAdV63-prime and MVA-boost (CM) regimen. The superior immunogenicity of this regimen is not unique to HIV immunogens, as it has also been demonstrated in preclinical and clinical studies of experimental malaria vaccines (Dunachie et al., 2006; Draper et al., 2010). Such repeated heterologous immunizations with the same transgene are known to increase both the magnitude and functional quality of vaccine-specific T cells and to allow more efficient migration to mucosal-associated tissues (Tatsis et al., 2007b). This is important in HIV infection, as effector immune cells in mucosal sites could block HIV transmission. It has also been shown that DNA priming followed with adenovirus boosting can reduce the level of anti-vector antibodies and increase transgene-specific immune responses (Xiang et al., 1999; Yang et al., 2003b), although this is questionable when considering the STEP study which employed a DNA-prime/Ad5-boost regimen. However, it is possible that this regimen effectively reduced the anti-vector antibody effect, thus curtailing a potentially worse outcome in the absence of DNA priming. Furthermore, prime-boost regimens with various combinations of adenovirus vectors were shown to induce robust frequencies of HIV-1 Gag-specific CD8+ T cells in nonhuman primates (Reyes-Sandoval et al., 2004), although it has to be appreciated that the level of pre-existing Ad5 immunity in NHPs would be lower or absent.

Adenoviruses are only associated with benign human pathologies, but their greatest limitation is pre-existing immunity which dampens vaccine-specific immunity by limiting transgene expression, while potentially exacerbating HIV acquisition. However, all else considered, Adenoviruses remain by far the most promising vaccine carriers for HIV-1, because unlike other vectors, they induce exceptionally high and persistent frequencies of vaccine specific T cells, which is a requirement for sustained HIV control. Although their efficacy has probably been hampered by high sero-prevalence, this no longer seems an insurmountable hurdle in light of the enormous amount of research efforts directed at finding strategies to circumvent the problems of pre-existing immunity (Gabitzsch et al., 2009). Additionally, replicating adenoviruses such as AdH4 and AdHu7 which can be delivered orally in the form of edible capsules might help to overcome pre-existing immunity (Xiang et al., 2003). Moreover, intranasal or oral delivery of adenoviruses has been shown to provide superior protection in animal models, and might trigger mucosal immune responses well-situated for preventing HIV acquisition. Perhaps adenovirus vectors engineered not to induce CD4+ T cells could be an alternative to overcome increased HIV-1 acquisition risk, although lacking CD4+ T cell help for the CD8+ T cells might compromise the differentiation and stability and thus efficacy of both CD8+ T cells and antibody responses (Yang et al., 2007b).

### Recombinant MVA (rMVA) vectors

Apart from their excellent safety profile, inherent adjuvant properties and ease of large scale production, recombinant vaccinia virus vectors are also popular for their large genomes which facilitate insertion of larger immunogens (Smith and Moss, 1983). MVA does not replicate in humans (Carroll and Moss, 1997) due to serial passaging in chick embryo fibroblasts which resulted in loss of more than 10% of its genome (Meyer et al., 1991), and its safety was well-documented during the smallpox eradication campaign (Mahnel and Mayr, 1994). MVA's potent immunostimulatory properties are achieved in a cascade of events involving induction of type 1 interferons, various chemokines for cell migration and activation of several cellular signaling pathways (Price et al., 2013). The immunostimulatory potency of MVA is largely attributed to the absence of genes involved in immune evasion (such as those that interfere with IFN-α, IFN-β, and TNF-α), thus allowing for stronger innate immunity to be generated (Antoine et al., 1998). MVA vectors are particularly important for generating strong T cell immunity against intracellular pathogens and cancers, but have also been shown to induce potent, high titre antibodies in a variety of disease models including SIV and malaria (Gherardi et al., 2003; Draper et al., 2008, 2013; Barouch et al., 2012). However, it is now well established that MVA vectors are more suited for boosting rather than priming, and depending on the priming vector (e.g., DNA or live vectors such as fowlpox and adenoviruses), MVA can induce various phenotypes of T cells, either predominated by CD4+ or CD8+ subsets or a combination of both.

In pre-clinical and clinical studies of malaria, recombinant MVA was shown to be highly immunogenic as it induced strong (and protective) cellular and antibody responses to malaria antigens, either on its own or when used to boost responses primed by vectors such as DNA, fowlpox or AdHu5 (Schneider et al., 1998, 1999; Gilbert et al., 1999, 2002; McConkey et al., 2003; Anderson et al., 2004; Webster et al., 2005; Bejon et al., 2007; Sheehy et al., 2011). Recombinant MVA85A (expressing the mycobacterial antigen Ag85A) was also shown to induce strong and durable T cell responses in various clinical studies (Scriba et al., 2012; Tameris et al., 2013, 2014). Furthermore, it was demonstrated that MVA expressing influenza A virus antigens (MVA-NP+M1) efficiently boosted CD8+ T cell responses to achieve clinical efficacy in humans (Berthoud et al., 2011; Lillie et al., 2012). As a therapeutic vaccine for cancer, recombinant MVA expressing the human papilloma virus antigens E2, E6, or E7, with or without IL-12 was shown to induce T and B cell immunity resulting in controlled HPV load and subsequent regression or complete elimination of precancerous lesions in a majority of vaccinees (Corona Gutierrez et al., 2004; Garcia-Hernandez et al., 2006; Albarran et al., 2007). Additionally, MVA expressing 5T4 antigen (TroVax) induced 5T4-specific antibody and cellular responses which correlated with tumor regression in a clinical trial of patients with advanced colorectal cancer (Harrop et al., 2006).

Although there is clear demonstration of the clinical efficacy of prophylactic and therapeutic MVA-vectored vaccines for malaria, TB, influenza virus and cancer, MVA vaccines for HIV are yet to be evaluated for clinical efficacy. However, Phase I and II studies of MVA expressing HIV antigens, either alone or in various prime-boost combinations indicate modest to strong immunogenicity (Guimaraes-Walker et al., 2008; Howles et al., 2010; Bakari et al., 2011; Garcia et al., 2011; Goepfert et al., 2011; Gomez et al., 2011). In particular, the MVA-B candidate HIV vaccine expressing monomeric gp120 and Gag-Pol-Nef poly-protein of clade B where MVA was administered without prior priming, induced long-lasting robust and polyfunctional effector memory T cell and antibody responses in Phase I/II studies (Garcia et al., 2011; Gomez et al., 2011). Furthermore, MVA has shown much higher immunogenicity when combined in prime-boost regimens with other priming vectors such as DNA, fowlpox or adenovirus (Goepfert et al., 2011; Keefer et al., 2011; Borthwick et al., 2014). In Phase 1 studies of the HIVA immunogen (based on HIV clade A and a string of CTL epitopes), priming with DNA (pTHr.HIVA) followed with MVA boosting (MVA.HIVA) was found to be immunogenic, inducing multifunctional and proliferative CD8+ and CD4+ T cell responses in greater than 70% of the vaccinees (Mwau et al., 2004; Goonetilleke et al., 2006).

As discussed earlier, a Phase I study combining DNA- and/or ChAdV63-prime followed with MVA boost to deliver an HIV-1 T cell immunogen induced high magnitude T cell responses with potent antiviral capacity (Borthwick et al., 2014). This study and similar studies of malaria vaccines (Sheehy et al., 2011, 2012; O'Hara et al., 2012) showed that the magnitude of T cell responses induced by ChAdV63 alone were modest, but significant boosting was achieved following MVA administration, thus highlighting the superior immunogenic potential of MVA when combined with appropriate priming vectors such as BCG (Whelan et al., 2009; Scriba et al., 2012), natural influenza A virus (Berthoud et al., 2011) or ChAdV63 (Colloca et al., 2012). Remarkably, a DNA/MVA prime boost of a vaccine expressing multiple HIV antigens induced responses in about 90% of volunteers and demonstrated strong immunogenicity despite pre-existing immunity to vaccinia virus (Sandstrom et al., 2008). As a therapeutic HIV vaccine vector, rMVA was found to be safe and to significantly augment HIV-specific CD4+ and CD8+ T cell responses in HAART-treated HIV-infected volunteers immunized with the MVA.HIVA candidate vaccine (Dorrell et al., 2006; Ondondo et al., 2006; Yang et al., 2007c). Furthermore MVA was found to be safe in neonates in a Phase 1 trial where MVA.HIVA was administered to infants born to HIV-infected or uninfected mothers (Afolabi et al., 2013). Therapeutic administration of MVA prime followed with fowlpox boost expressing Env, Gag, Tat, Rev, and Nef-RT fusion antigens increased the frequencies and breadth of T cell responses in young adults (Greenough et al., 2008).

One very attractive feature of rMVA (and other poxvirus vectors) is their ability to induce mucosal immune responses when administered via mucosal routes (Gherardi and Esteban, 1999, 2005). In particular, murine and macaques studies using rMVA vectors demonstrated induction of protective HIV-specific immune responses within the genito-rectal mucosae, which in some cases correlated with reduced disease progression (Belyakov et al., 1998a; Makitalo et al., 2004; Wang et al., 2004). Enhanced immunogenicity of rMVA in combination with DNA priming was also achieved by using the non-toxic B subunit of cholera toxin (CTB) as mucosal adjuvant (Gherardi et al., 2004). Thus, even though MVA may be inadequate as a stand-alone delivery platform, it definitely shows greater potential as a boosting vector (especially for the chimpanzee adenoviruses) and should be evaluated for efficacy in advanced HIV vaccine trials.

### Recombinant NYVAC vaccine vectors

NYVAC vector is also a vaccinia-based vector which was highly attenuated by deletion of 18 genes involved in host range virulence. It has been shown to induce mainly CD4+ T cell responses, in contrast to MVA which has a stronger immunostimulatory potential and is known to induce both CD8+ and CD4+ responses (Mooij et al., 2008). However, in a trial of chronically infected patients on HAART, a NYVAC-based vaccine expressing Gag/Pol/Nef/Env from an HIV-1 clade B isolate (NYVAC-B) was found to be highly immunogenic and induced high magnitude, broad and polyfunctional CD4+ and CD8+ T cells (Harari et al., 2012). Similar to MVA, NYVAC elicits greater immune responses when used in prime-boost combinations rather than on its own (Harari et al., 2008; McCormack et al., 2008). In these EuroVacc studies, priming with DNA-C followed with NYVAC-C boost elicited broad, polyfunctional and durable CD4+ T cell responses in greater than 90% of volunteers, compared to only 40% when NYVAC was used alone (Harari et al., 2008). Moreover, in a preclinical study with a DNA prime followed with NYVAC boost, responses to a vaccine expressing SIV-Gag/Pol/Env were boosted 10-fold with improved quality and quantity of T cell responses (Hel et al., 2001). A NYVAC/SIV-gpe vaccine (expressing SIV Gag/Pol/Env) also elicited mucosal immune responses in macaques following both mucosal and systemic delivery (Stevceva et al., 2002). Despite the skewing toward CD4+ T cell responses, NYVAC has potential to stimulate and boost more balanced immune responses when combined with other vectors, and its potential should be fully explored, especially for therapeutic HIV vaccines which require re-invigoration of CD4+ T cell functions (and frequencies).

### Canarypox (ALVAC) vaccine vectors

ALVAC is an attenuated derivative of the canarypox virus that was repeatedly passaged in chick embryo fibroblasts and thus has restricted tropism with very minimal pathogenicity in humans (Yu et al., 2006). Despite the comparatively lower immunogenicity with respect to other poxvirus vectors such as MVA (Zhang et al., 2007) and NYVAC, the fact that ALVAC has no potential pre-existing immunity in humans makes it a more attractive HIV vaccine delivery vector. The ALVAC vector (vCP205) was shown to be safe and to induce strong CD8+ CTL and antibody responses to an HIV vaccine expressing gp120/41 and Gag/Pol sequences [ALVAC-HIV(vCP205)] in a Phase 1 clinical trial in the USA in the 1990 s (Belshe et al., 1998). A related ALVAC-based vaccine expressing multiple HIV antigens comprising Gag, Env, Nef, Pol and Pro [ALVAC-HIV(vCP300)] also induced durable CTL responses in healthy volunteers (Evans et al., 1999). In preclinical studies, ALVAC expressing SIV Gag/Pol/Env protected against low-dose oral SIVmac251 challenge of neonate rhesus macaques in a study design aiming to mimic HIV transmission through breast milk (Van Rompay et al., 2005). More recently ALVAC-based HIV vaccines have been tested in both adults and infants, where they have shown modest immunogenicity (Kintu et al., 2013; Kaleebu et al., 2014) and in the RV144 trial of ALVAC prime [ALVAC-HIV(vCP1521)] and protein boost (AIDSVAX B/E rgp120), the only HIV vaccine candidate to show efficacy (Rerks-Ngarm et al., 2009, 2013).

While it is unclear whether the modest success of RV144 was due to the immunostimulatory potential of canarypox virus vector or immunogenicity of the vaccine inserts, the fact that the immunogens in the RV144 trial vaccines are not significantly distinct from those used in other HIV vaccines in the field eliminates the “immunogen effect,” thus leaving the vectors and delivery methods as possible explanations. But, as the AIDSVAX vaccine (recombinant gp120) showed no efficacy in earlier trials (VAX003 and VAX004), the success of RV144 points to the delivery vector (ALVAC) and possibly the benefits of a combined viral vector and protein immunization regimen as opposed to homologous boosts. This might suggest that combined live vector-priming and protein-boost immunization modalities could be further refined to achieve greater potential for increased efficacy. Alternatively, protection by the combined vaccines could be attributed to T cell help for the antibody responses. It must however be noted that unlike the RV144 study, VAX003, and VAX004 were conducted in high-risk populations, which might be a strong confounding factor, although this might as well be reflective of the very limited efficacy of stand-alone protein subunit vaccines for HIV. Despite the modest efficacy of RV144, the immune responses waned within a short time indicating that ALVAC may not be a particularly suitable vector to induce long-lived anti-HIV immunity, unless it is combined with other powerful vectors. In direct comparison of immunogenicity, ALVAC was found to be less immunogenic than MVA, possibly due to MVA's enhanced antigen expression within dendritic cells (Zhang et al., 2007). Nonetheless, ALVAC is still quite promising for HIV vaccine delivery, as it is also already licensed for delivery of several veterinary vaccines including the feline leukemia virus (FeLV) and feline rabies vaccine (PUREVAX) and RECOMBITEK vaccine which protects against canine distemper, equine influenza and West Nile Virus.

## Mycobacterium bovis bacillus calmette-guerin (BCG) vaccine vectors

Prevention of breast milk transmission of HIV-1 remains an important goal for HIV vaccine researchers. BCG is an attenuated vaccine proven to be safe and has for many years been administered to new-born babies to immunize against Mycobacterium tuberculosis (Mtb). As such, BCG provides a platform to co-deliver HIV immunogens in neonates to potentially protect against mother-to-child transmission of HIV-1. The potential use of BCG as an HIV vaccine vector was explored in preclinical studies of adult and new-born BALB/c mice using the HIV-1 clade A Gag immunogen (HIVA) (Mwau et al., 2004). Priming with recombinant BCG expressing HIVA (BCG.HIVA) induced HIV-specific T cell responses which were efficiently boosted with rMVA (MVA.HIVA) (Hopkins et al., 2011a,b; Saubi et al., 2011, 2012). In further related studies, priming with BCG.HIVA and boosting with a combination vaccine expressing HIVA and the Mtb antigen 85A (mMVA.HIVA.85A) induced robust IFN-γ-producing T cells to both HIV-1 and Mtb antigens. Moreover, in adult mice, BCG.HIVA primed weak HIV-1-specific CD8+ T cell responses, which were strongly boosted with either Ad5 (HAdV5.HIVA) or rMVA (MVA.HIVA). Thus, immunization of neonates with recombinant BCG expressing HIV-1 immunogens, followed with an MVA boost expressing the same HIV immunogen might concurrently protect against Mtb and HIV-1. It remains to be seen how these rBCG-vectored HIV-1 vaccines will perform in clinical studies.

## Replication-competent viral vectors

The unprecedented success of the SIVmac239 Δnef experimental vaccine in rhesus macaques (Reynolds et al., 2008, 2010) gives a hint that possibly, a successful HIV vaccine will require a live delivery vector, as these are known to induce high magnitude, durable and broadly effective immunity. But as exciting as this may sound, there are significant challenges in terms of balancing the safety and immunogenicity vs. replicative capacity. Of the adenoviruses, Ad4 and Ad7 have been tested in clinical studies (by oral delivery) and were successfully used for the prevention of respiratory and enteric illnesses (Hoke and Snyder, 2013). These replication competent adenoviruses naturally infect and replicate in mucosal tissues (Patterson and Robert-Guroff, 2008) and could thus be quite relevant for HIV vaccines. Preclinical studies of recombinant Ad4 expressing HIV-1 clade C envelope gp160 (Ad4Env160), gp140 (Ad4Env140), and gp120 (Ad4Env120) demonstrated induction of envelope-specific T cells in mice and antibody responses in rabbits (Alexander et al., 2013). Serum from the rabbits was able to neutralize a tier 1 clade C pseudovirus and to a lesser extent, homologous and heterologous tier 2 pseudoviruses.

A replicating CMV vectored SIV vaccine (RhCMV-SIV/Gag, Rev/Nef/Tat, Pol, Env) was shown to persist in vaccinated rhesus macaques and conferred durable protection from disease progression owing to induction of high magnitude effector memory CD8+ T cells, despite pre-existing CMV immunity (Hansen et al., 2009, 2011, 2013). Other replication-competent viruses in clinical development include the TianTan vaccinia virus (TT), Vesicular stomatitis virus (VSV), a derivative of NYVAC (NYVAC-C-KC) and Sendai virus (SeV). The TianTan vaccinia virus was used in a DNA-prime (pCCMp24)/Tiantan boost (rddVTT-CCMp24) regimen where it was shown to induce antibody and HIV-specific T cell responses (including memory phenotypes) following intramuscular delivery and has now been advanced to Phase II clinical study in China (Excler et al., 2010; Liu et al., 2013). The NYVAC-C-KC vectors have shown superior cellular and humoral immunity compared to the non-replicating NYVAC, at least in mice (Kibler et al., 2011; Gomez et al., 2012).

A Sendai virus vector expressing SIV Gag (SeV-Gag) administered intranasally as a boost following intramuscular priming with an envelope-independent DNA vaccine (CMV-SHIVdEN) demonstrated very strong suppression of intravenous SIVmac239 challenge in rhesus macaques, which was extended over a 3-year period (Matano et al., 2001; Takeda et al., 2003; Kawada et al., 2007). Clinical investigations of a SeV-based candidate HIV vaccine expressing Gag [SeV-G (NP)] are ongoing in Rwanda, Kenya and the UK, and it is expected that results of these trials will provide a feel of the potential of Sendai virus as an HIV vaccine vector. Attenuated VSV is a non-pathogenic, low sero-prevalence vector that was also found to be quite promising as it achieved virus control during SHIV89.6P challenge experiments in rhesus macaques immunized with rVSV expressing Gag and Env (Rose et al., 2001). Recombinant VSV vector was shown to induce strong memory CTL responses to HIV-1 Gag and Env in mice, which were significantly amplified by boosting with heterologous recombinant vaccinia virus vectors (Haglund et al., 2002). It is postulated that intranasal delivery of rVSV vaccines in combination with IL-12 administered during DNA priming may elicit mucosal immunity for HIV (Egan et al., 2004, 2005). Priming with rVSV-Gag/Pol/Env (VSV-SIVgpe) followed with MVA-Gag/Pol/Env (MVA-SIVgpe) boost was shown to induce strong and long-lived antibody and cellular responses that achieved long-term control of SHIV replication (Schell et al., 2009; Van Rompay et al., 2010). An ongoing phase 1 trial of rVSV-HIV-1 Gag vaccine (HVTN090) has demonstrated clinical safety and T cell immunogenicity following intramuscular delivery (Fuchs et al., 2012, 2013), although the magnitude of responses was limited and will most likely require priming (or boosting) with suitable vectors.

Other vectors being explored include rhadinovirus (Bilello et al., 2011), yellow fever virus (Bonaldo et al., 2010), rabies virus (Faul et al., 2009), Venezuelan equine encephalitis virus (VEEV) (Caley et al., 1997) and Semliki Forrest virus (Schell et al., 2011), all of which have shown strong immunogenicity, with some achieving efficacy in NHP challenge protection models. Influenza virus vaccine vectors have also been studied extensively and have been successfully used as delivery vehicles for several experimental HIV vaccines (Li et al., 1993a, 2013; Muster et al., 1994, 1995; Garcia-Sastre and Palese, 1995; Palese et al., 1997; Sexton et al., 2009). As natural mucosal pathogens, influenza virus vectors are well-adapted for stimulating robust mucosal and systemic immunity comprising both antibody and cellular immune responses (Garcia-Sastre and Palese, 1995; Palese et al., 1997; Li et al., 2013). Mucosal immunization of mice with chimeric influenza virus vectors expressing the HIV-1 gp120 V3 loop peptide (IHIGPGRAFTYTT) (Li et al., 1993a) or the gp41 epitope (ELDKWA) (Muster et al., 1993, 1994, 1995) was shown to induce persistent antibody and CTL responses. Influenza virus vectors might be successfully combined in prime-boost regimens as demonstrated in influenza virus-prime and MVA-boost studies in mice (Gherardi et al., 2003), although they have a limited capacity for immunogen insertion.

## Heterologous prime-boost strategies for enhanced HIV vaccine efficacy

Repeated vaccination in heterologous prime boost approaches employing different vector combinations in a specific order is widely accepted as the most efficient means to induce superior quality and quantity of vaccine-specific immune responses (Li et al., 1993b; Ramshaw and Ramsay, 2000; Estcourt et al., 2002; McShane, 2002; Newman, 2002). Heterologous prime boost regimes allow immune boosting without creating problems of anti-vector immunity. Furthermore, heterologous prime-boosts result in increased frequencies of memory T cells, and it has been shown that the number of immunizations can significantly influence the phenotype of vaccine-specific memory T cells, with secondary and tertiary immunizations generating effector-like memory T cells which preferentially accumulate in non-lymphoid organs (Masopust et al., 2006; Nolz and Harty, 2011). These findings have huge implications on the quality and potential of mucosal surveillance of cells induced in prime-boost vaccination protocols.

Distinct live viral vectors can be combined in prime-boost regimes to maximize immune responses. In most studies DNA has been used for priming, but recently a number of virus vectors including Adenoviruses, influenza viruses as well as fowlpox and canarypox have been tested in prime-boost regimens. Prime-boost regimens comprising Adenovirus and MVA or heterologous Adenovirus strains have recently been shown to induce both cellular and humoral immune responses to SIV and malaria antigens (Draper et al., 2008; Liu et al., 2009; Tatsis et al., 2009; Barouch et al., 2012). In particular, impressive protection against SIV acquisition in rhesus monkeys was achieved following immunization with a SIV_SME543_-Gag/Pol/Env vaccine delivered by Ad26/MVA and Ad35/Ad26 prime-boost regimens which induced a mixture of neutralizing and binding antibody as well as cellular immune responses (Barouch et al., 2012). This study further demonstrated induction of both systemic and mucosal immune responses and achieved protection from both acquisition and disease progression, thus providing proof of concept that HIV-1 acquisition and post-infection control might be achieved by improved immunogen design and delivery strategies. Heterologous or homologous regimens comprising DNA/MVA, MVA/Ad26, and MVA/MVA were comparatively less efficacious than Ad26/MVA or Ad35/Ad26, which reduced viral load set-points by greater than 100-fold. A Phase 1 clinical trial (B003/IPCAVD-004) assessing the immunogenicity of various prime-boost combinations of Ad26 and Ad35 is ongoing, and will inform the field on the clinical utility of these two promising human adenovirus vector combinations. Another NHP study employing three doses of plasmid DNA followed with Ad5 to deliver various immunogens comprising SIV-Gag, SIV-Env mosaic immunogens or SIV_mac239_ Env also induced cellular and antibody responses (neutralizing antibodies and ADCC) and achieved significant protection against intra-rectal challenge of rhesus macaques with SIV_smE660_ that was a mismatch of the vaccine strain (Roederer et al., 2014). Moreover, superior immunogenicity of prime-boost combinations using DNA/ChAdV63/MVA or ChAdV63/MVA has been demonstrated in a Phase I study (Borthwick et al., 2014).

The success of a viral vector for priming has already been demonstrated in the RV144 study which used ALVAC to prime antibody and T cell responses, followed with a protein boost (Rerks-Ngarm et al., 2009). Although priming with DNA has always seemed a better strategy as it focuses the immune response to the immunogen transgene, as opposed to viral vectors which carry multitudes of immunogenic antigens within their backbones, the efficacy of viral-vector priming followed by protein boosting in the RV144 study and the superior immunogenicity of virus-prime/virus-boost in the studies discussed above support the use of viral vectors for both priming and boosting. Therefore, heterologous prime-boost regimens combining DNA, Adenovirus and MVA or ALVAC are likely to achieve efficacy against HIV in clinical trials, although this will require that HIV Env or genes encoding NAb epitopes are included in the immunogen formulations (Barouch et al., 2012, 2013). Preclinical studies investigating the potential of combined chimpanzee adenovirus, MVA and protein prime-boost regimens to deliver immunogens which can stimulate broadly neutralizing antibodies such as BG505 are underway. The success of recombinant adenovirus vector priming followed with MVA boost in inducing high-titre antibodies either on their own or in conjunction with molecular adjuvants has already been proven in preclinical studies of malaria (Draper et al., 2008). Possibly the persistence of adenovirus ensures continuous antigen supply which is suitable for B cell priming. It is envisaged that optimal delivery modalities which combine HIV immunogens eliciting BNAbs with those that stimulate strong T cell immunity will achieve enhanced vaccine efficacy. Of course a major caveat of combining strong T cell vectors with antibody-producing immunogens is the possible immune interference of antibody production by these vectors. Nevertheless, this can be optimized perhaps by employing several protein boosts with powerful adjuvants in order to deliver the most balanced immune responses.

## Potential vaccine-associated risk of HIV acquisition

The increased risk of HIV-1 acquisition in the STEP and HVTN505 trial vaccinees despite strong immune responses has raised many unanswered questions as to whether the vaccine delivery modalities, suboptimal potency of the HIV immunogens or other unknown external factors are responsible for vaccine failure. As far as immunogen design, the vaccine construct used in the STEP, Phambili and HVTN505 studies represents one of the most comprehensive immunogens with broad coverage, as it comprised a 6-plasmid DNA and rAd5 vectors expressing Gag/Pol/Nef/Env proteins from multiple clades. Other immunogens based on similar or far less comprehensive HIV protein coverage have also been tested and showed varied degrees of immunogenicity. Thus, an understanding on whether the outcomes of the STEP/Phambili/HVTN505 studies (efficacy, immunogenicity or increased risk of acquisition) would have been different if other delivery vectors (such as DNA/MVA, DNA/ALVAC or DNA/Ad35/Ad26 or even a replicating CMV vector) had been used to deliver the same immunogens in these trials is key for further progression in the field. An Alternative way to look at this is to ask whether the results of RV144 trial would have been worse if Ad5 was used instead of ALVAC, assuming that the prevalence of Ad5 neutralizing antibodies in the RV144 population does not differ significantly from the STEP and Phambili study populations.

The finding that the vaccine was not at all efficacious amongst men who were circumcised or in uncircumcised men who did not have pre-existing Ad5 immunity raises doubts as to whether efficacy was genuinely hindered by Ad5 serostatus. This is further supported by the results of HVTN505 study which tested only circumcised individuals without Ad5 antibodies, yet no protection was observed. Moreover, the absence of Ad5 antibodies in the HVTN505 study participants (which should in theory allow for higher immunogenicity) was not associated with any significant enhancement of the magnitude and quality of immune responses over those seen in the STEP and Phambili studies. Therefore, Ad5 serostatus can be safely removed from the equation, leaving the only plausible explanation for vaccine failure to be the quality and quantity of immune responses. If this can be fully documented beyond doubt then it implies that either the Ad5 delivery vector or the HIV-1 antigens used were not immunogenic enough to afford protection from infection or post-infection virus control. However, considering that Ad5 is one of the most immunogenic vectors currently available, (and that the immunogen used in these studies was comprehensive and well-designed), this would have serious implications for vaccine design, as it sets the bar really high for new candidate vaccines which would be expected to stimulate responses of extremely higher magnitudes and superior qualitative properties in order to achieve even the minimal efficacy. On a brighter side, this would perhaps instigate intense scrutiny of the current methods used for assessing vaccine immunogenicity in order to standardize and synchronize with those for efficacy measurements.

One other interesting question is whether (and how) Ad5 sero-positivity is intrinsically associated with HIV acquisition. Although studies of uncircumcised men document increased risk of natural HIV acquisition due to a high frequency of CD4+CCR5+ target cells in the foreskin (Prodger et al., 2012), how this relates their Ad5 sero-positivity and titre levels with infection risk is not very clear. However, the fact that the risk of HIV-1 acquisition in the STEP study diminished with time after immunization, and eventually leveled up with placebo recipients (Buchbinder et al., 2008) might in actual fact support a role for vaccine-induced immune activation in HIV acquisition (Tenbusch et al., 2012). Perhaps this could be as a result of generalized immune activation or induction of activated vaccine-specific HIV-1 targets with mucosal-homing properties. Should this be the case, then this would not be unique to Ad5 vectors alone and it would therefore be expected to equally affect other delivery vectors capable of inducing activated mucosal-homing target cells. However, as there were no notable differences in activated circulating T cells between vaccinees and placebos, it is unlikely that generalized vaccine-induced immune activation played a role, although it remains possible that there could have been significant differences in activated targets at mucosal sites which were not measured.

This then raises another interesting question as to whether the outcome of the STEP/Phambili/HVTN505 studies would have been significantly worse (or better) had the vaccines been administered mucosally. This question might have two sides to it, in the sense that mucosal delivery would probably have generated higher frequencies of activated HIV targets at the genital mucosae, hence increasing the potential of fuelling infection. On the other hand, induction of robust and polyfunctional effector immune responses at mucosal portals of HIV entry would probably have cleared the incoming HIV before infection became established. Although these questions have no clear cut answers and cannot be addressed retrospectively in the context of the clinical trials they relate to, they however highlight the extreme challenges in HIV vaccine delivery, and new studies designed to directly tackle these issues will be quite informative for future vaccine development research. Studies looking at whether the most promising delivery vectors (and the respective immunogens) can concurrently induce activated HIV-1 target cells that preferentially home to and persist in the genito-rectal and GALT mucosae, and whether or not such vaccine-induced cells become highly permissive to HIV infection will be of particular interest in efforts aimed at limiting the risk of vaccine-induced HIV-1 acquisition and accelerated disease progression.

## Perspectives and conclusion

Ideally, vectors for HIV-1 vaccines should directly target antigen presenting cells (APCs) or other immune cells to induce long-lived, strong antibody and cellular responses that can broadly disseminate to systemic and mucosal compartments. The vaccine-specific T cells in particular should be broad and contain activated effector, effector memory and central memory phenotypes in various proportions in order to achieve a proper balance between immediate virus clearance and sustained immune-surveillance for long-term protection, as demonstrated by the RhCMV-SIV vaccine which controlled and cleared pathogenic SIV infection (Hansen et al., 2009, 2011, 2013). Furthermore, vectors which can stimulate polyfunctional CD4+ and CD8+ T cells that act in concert with B cells to inhibit HIV replication through a variety of mechanisms would be more successful than those inducing only mono-functional T cells of either subset alone.

Of particular relevance to protection from infection would be vaccine vectors associated with homing and long-term persistence of vaccine-induced immune responsive cells at the genito-rectal mucosae (Chanzu and Ondondo, 2014) as well as other mucosal sites serving as HIV reservoirs. This remains a very important priority in consideration of the significant rapid CD4+ T cell depletion in the intestinal mucosa despite successful HAART (Brenchley et al., 2004; Mehandru et al., 2004). Thus, vaccine vectors which naturally infect cells within mucosal inductive sites, especially the replication-competent viruses such as adenovirus and influenza virus vectors (Gherardi et al., 2003; Sexton et al., 2009) which can be administered mucosally to trigger mucosal immunity, would be more suited for HIV vaccine delivery. Alternatively, delivery of vaccines via routes which enhance mucosal immunity (Holmgren et al., 2003; Holmgren and Czerkinsky, 2005; Czerkinsky and Holmgren, 2012) or vectors possessing an inherent ability to induce mucosal immunity in addition to systemic immune responses following parenteral or mucosal vaccine delivery (Moser et al., 2007) may be employed. Virosome vectors for instance, possess intrinsic adjuvant properties and a unique ability to target antigen presenting cells, hence have been very successful at inducing protective mucosal immunity in SHIV challenge models (Moser et al., 2007; Bomsel et al., 2011; Leroux-Roels et al., 2013). Other vectors suitable for mucosal vaccine delivery include VEEV (Caley et al., 1997). In the absence of mucosal delivery vectors, new delivery technologies such as the “prime and pull” approach may be utilized in conjunction with systemic delivery methods to enhance mucosal homing and subsequent immunity (Azizi et al., 2010; Shin and Iwasaki, 2012; Tregoning et al., 2013). In this approach, specialized chemokines are administered in mucosal compartments following parenteral immunization in order to chemo-attract the activated vaccine-specific immune cells from the systemic compartments. Furthermore, use of mucosal adjuvants such as CTB and LT-B (Albu et al., 2003; Yuki and Kiyono, 2003), pro-inflammatory cytokines (IL-1α, IL-12, and IL-18) (Belyakov et al., 1998b; Bradney et al., 2002; Albu et al., 2003) or immunostimulatory CpG motifs (Horner et al., 2001; Dumais et al., 2002; Daftarian et al., 2003; Jiang et al., 2005) which target recruitment of immune cells to the mucosal sites would be useful. Co-delivery of vaccines with genes encoding CCL19 and CCL28 was also shown to enhance HIV-1-specific T and B cell responses in the systemic as well as mucosal compartments (Hu et al., 2013).

In consideration of both safety and immunogenicity goals as already discussed, and with particular emphasis on the pivotal role of CTL responses in controlling HIV replication, it seems that non-replicating viral vectors with lower sero-prevalence would be highly desirable, mainly due to excellent safety profiles and potent adjuvant effect allowing for induction of very strong, high quality and long-lived cellular and humoral immunity. However, although safety and reduced immune interference would be guaranteed, a major caveat would be that these lower sero-prevalence vectors may not be adequately immunogenic. Perhaps these vectors can be re-engineered to improve their immunogenic potential. For instance, the immunogenicity of vectors such as MVA and NYVAC can be improved by removal of genes associated with immune evasion which counteract immune responses to the vaccine (Kibler et al., 2011; Gomez et al., 2012; Garcia-Arriaza et al., 2013). In other cases, addition of cytokine-encoding genes such as type 1 interferons, IL-12 or GM-CSF can enhance vaccine efficacy (Gherardi et al., 1999, 2000; Rodriguez et al., 1999; Ramshaw and Ramsay, 2000; Bayer et al., 2011). Furthermore, chemokines such as CCL3 which recruits professional APCs can be co-delivered with HIV antigens to enhance vaccine immunogenicity (Lietz et al., 2012).

Alternatively, vectors capable of inducing substantial immunogenicity in the presence of pre-existing natural or vaccine-induced anti-vector immunity may be worth considering, although it is expected that finding highly attenuated vectors which are safe and remain immunologically potent will be equally challenging. As discussed earlier, combining some of the most promising vectors in heterologous prime-boost regimens will significantly enhance the quantity, quality and protective efficacy of immune responses. However, in consideration of the possible catastrophic effects of elevated immune activation likely to arise from various vector combinations, it would be expected that suitable HIV vaccine vectors maintain lower levels of immune activation to limit the numbers of activated HIV-1 targets (Perreau et al., 2008; Benlahrech et al., 2009) likely to fuel infection in the event of exposure. Furthermore, it is documented that in the absence of a very strong protective immune responses to counteract the incoming virus, the presence of vaccine-specific T cells which are activated and hence more susceptible to infection may increase the risk of acquisition (Tenbusch et al., 2012). Whether it is possible to achieve potent immunostimulatory capacity but with minimal immune activation still remains a subject of intense investigation.

When safety and versatility are considered, and in full view of the enormous technology advancements in DNA plasmid formulations and delivery, in conjunction with other immunomodulatory interventions such as SAP depletion and use of molecular adjuvants, recombinant DNA vaccines remain very attractive, although efforts to improve stimulation of long-lived effector/memory CD8+ T cell phenotypes are still needed to achieve long-term efficacy. Undoubtedly, repeated immunizations or combining DNA vaccines with persistent (replicating) vectors or vectors with slow immunogen release features would induce durable immunity. Nonetheless, replicating vectors with lower sero-prevalence and minimal pathogenicity (Rose et al., 2001; Kawada et al., 2007; Fuchs et al., 2013; Liu et al., 2013) are being considered as they would provide a persistent pool of HIV vaccine-specific effector memory phenotype cytotoxic T cells which are critical for long-term protection from disease progression (Hansen et al., 2009, 2011, 2013). Such effector memory responses would otherwise be expected to wane with time, in the absence of antigen. Replicating vectors may also be better-suited for induction of broadly neutralizing antibodies since persisting expression of the Env antigens is likely to drive high levels of somatic mutations required for affinity maturation of these antibodies (van Gils and Sanders, 2013). A new strategy that has been proven to induce durable and protective antibody responses in humanized mice challenged with high doses of diverse HIV strains is vectored immunoprophylaxis, which involves insertion of immunoglobulin genes into viral vectors such as the adeno-associated virus (AAV) to provide long-term expression of neutralizing antibodies (Balazs et al., 2012, 2014). Moreover, inclusion of Th2 cytokines such as IL-4, IL-5, and IL-6 which enhance B cell maturation into long-lived antibody secreting cells is yet another strategy already shown to induce high titres of neutralizing antibodies which protected mice from Friend Virus (Ohs et al., 2013). Other possible strategies include use of lentiviral vectors expressing B cell receptor genes encoding neutralizing antibodies to HIV-1 to transduce haematopoietic stem cells (Luo et al., 2009).

Since optimum induction of immune responses to vaccines strongly depends on innate immune triggering as well as the levels of transgene expression, vectors with natural adjuvant properties and therefore capable of strongly inducing innate immunity are particularly immunogenic and thus highly desirable. However, care must be taken to balance between strong innate function stimulation and the potential risk of inducing potent stimulation of immuno-pathological effects, including immune hyper-activation.

In conclusion, a successful vaccine for HIV will have to stimulate potent antibody and CTL responses broad enough to cover multiple HIV variants and with potential to neutralize, bind or suppress HIV-1 replication for sustained (possibly infinite) lengths of time. Of utmost importance, however is generation of vaccine-specific immune responses in the genito-rectal mucosae, the major portals of HIV entry. Emerging evidence strongly suggests that non-pathogenic, low-level replicating viral vectors which can mimic live attenuated vaccines, but with low sero-prevalence might be the best way to achieve HIV vaccine efficacy. As these vectors persist long after immunization, they are capable of inducing and maintaining effector/memory CTLs for continued immune surveillance that is necessary to protect from infection, disease progression and to clear or prevent establishment of latent reservoirs. Thus, to achieve protective efficacy HIV vaccine development will need ingenious state of the art technologies to create the very best of T cell and antibody immunogens, delivered by the most potent but safe vectors possessing remarkably high capacity to induce both systemic and mucosal immunity, but without significant immune activation likely to fuel HIV acquisition. Recent significant advances in vaccine delivery technologies and HIV immunogen design provide hope that this is not far from reality.

### Conflict of interest statement

The author declares that the research was conducted in the absence of any commercial or financial relationships that could be construed as a potential conflict of interest.
